# Mental Influence in Physical Disease

**Published:** 1869-02-01

**Authors:** John Williamson

**Affiliations:** Chicago


### CHICAGO MEDICAL JOURNAL.

VOL. XXVI.—FEBRUARY 1, 1869.—NO. 3.

### ORIGINAL COMMUNICATIONS.

### *Mental Influence in "Physical" Disease.*

AN INAUGURAL THESIS FOR THE DEGREE OF M. D., BY JOHN WILLIAMSON, CHICAGO.

To medical science, more than to any other, is all knowledge tributary. First in importance to man, it is first in facility of attainment and diversity of fulness. In medicine no culture comes amiss, no zeal is unrequited. The possibilities of its empire are infinite, its present exaltation a marvel. Its catholic spirit is a sure insignia of conquest, its victories are its sublime apology. Its spirit of rational and positive inquiry, associated with its heroic self-abnegation, have been in all time the terror and admiration of the human mind. There can be but one Medical Science, as there can be but one science of Chemistry, Geology or Geometry. Opinion here is inadmissable; it is not what a man *believes*, but what he can *prove*, that is valuable in medicine. Before the rigorous application of the philosophy of induction to the determination of all truth, the unaided effort of reason was deemed competent to unfold and exemplify all that it was important or desirable to know. While induction is the only possible means of scientific investigation, the existence and cöordination of its factors—reason and observation—are not to be denied. The relation of the first to medicine, this essay proposes to investigate. Preconceived opinion, in almost every instance, is allowed to influence experiment. But few are willing to let so unassuming a messenger as science correct elaborate and learned ignorance and conceit. Our opinions, of long standing especially, are our own property, and occupy in consequence a distinguished place in our esteem, while truth being the heritage of all can never be appropriated exclusively.

What I propose in this paper is simply an inquiry. That the mind and body do reciprocally influence, in some degree at least, is indisputable. Concerning the quantity and extent of this influence opportunity exists for interesting and instructive controversy and research.

That there may be no mistake relating to my terms and logic, a few preliminary definitions are advisable.

If my phraseology and reasoning are inaccurate in facts, and unscientific in philosophy, I trust their impress of personal subjectivity may be an apology for the blunders, and excuse the instinctively evoked criticism.

A scientific truth, is the fundamental and, in consequence, unavoidable response to the question, Why?

A science, is the systematized association of these fundamental responses.

By natural, I mean that which is consistent with the actualties or possibilities of experience.

Experience is the sum of mental exercises, and knowledge is the sum of experience.

All force is phenomena, and all phenomena are force.

All motion is force, and all force is motion: then all motion is phenomena, and all phenomena are motion.

Such dogmatism as this is not allowed, but by postulates.

Philosophy is the investigation of the relations of facts and forces.

By law is meant that relation existing between phenomena by which their mutual consanguinity is established.

Life is affirmable only of the physical; if there be in man any spiritual quality, it is not "alive" but conscious. Consciousness is the "life" of the mind. Every distinct mental activity is a faculty.

Matter is the summation of correlated force, whose properties are partly active and partly potential. Mind is an element in this summation, generally potential, but vigor, ously actual under appropriate conditions of organization. The quantitative identity of all force being conceded-there can be no dispute concerning the qualitative sympathy, and thus is afforded an emphatic intimation of mental power influencing material change. When this material change is abnormal it constitutes disease.

No exercise of force is possible without molecular waste. Mentality furnishes no exception to this rule. No increment of thought is possible without being induced by and inducing palpable tissue metamorphosis. To this psycho-physiological law there is no exception. All force, so far as we know, has its origin in the solar ray. Solar power is potentially lodged in diet, and becomes actual in organization. Organization is the legitimate and invariable sequence of life. It may be termed the expression of life. By many life is regarded as merely the dignitary presiding over the vital functions. It is more than this; it is the necessary antecedent, determining organization in general, and likewise every specific function. Life and organization, in the present condition of our knowledge, are the constant conditions of mentality. Should it be thought impossible then, or indeed, even strange, that mind influencing life should determine, in very many cases, structural lesion. Should not an intimate and earnest acquaintance with the science and relations of mind, be regarded as an indispensible auxiliary to practical therapeutics.

Other things being equal, mental, moral and physical power are determined by the quantity and quality of food eaten. This unity of origin is powerfully suggestive of a common dependence, and dynamic concord and sympathy. I assume in this essay that inductive reasoning is philosophically composite, involving induction proper, by which generalizations are justified, and deduction, affirming of an individual what the generalization affirms of the class to which the individual belongs. My use of these essential factors being understood, no confusion of logic need exist.

In the inquiry I am making I would not ignore the value and facility of hypothesis. It is a ready and convincing element in all inductive reasoning. Without it science would be at a stand-still. Conclusions are never born naturally, they are creations of cultivated art. The only natural element of science, is the essential and antecedent hypothesis. An hypothesis is not a mere guess, as most people without reasoning conclude. In what follows its definition may be found. The "known" and "unknown" are relative terms, distinguishing human knowledge. The "known" is actual, the "unknown" possible revelation. It is from the "unknown" that the "known" is recruited and extended. There is no impenetrable barrier separating these grand kingdoms. The bright rays of hypothesis perpetually maintain a cheerful radience in the otherwise profound and hopeless gloom. Hypothesis is the mind's rational, scientific judgment of the "unknown," guided by the light of demonstration. The too current practice of interchangeably using hypothesis and assumption, as indiscriminate synonyms, cannot be too earnestly deprecated by every friend of an exact and versatile nomenclature. I may be excused for my lengthy allusion to this subject, since the scientific philosopher finds no greater impediment to remove, than this unintended or malicious confounding of terms and meanings. Before the mariner touches the shore, all on board are made glad by his ringing cry, "land ahead!" So the man of science, standing upon the brink of the "known," distinctly sees truth "yet to be." This is hypothesis.

No organ of the body can be normally exercised without appropriate stimulation. Pure blood for the heart, air for the lungs, portal blood for the liver, nitrogenized aliment for the stomach, chyme for the intestines, chyle for the lacteals, etc., are the law of life. The brain, like the heart and lungs, is an organ whose function is to think. Like them, it too must be aroused and sustained by stimuli. These are external impressions. If this stimulation be denied or but indifferently supplied, sleep first, and unless relief speedily come, death unfailingly supervene. Thought then, is impossible without external impressions. Memory, as a type of mental action, beautifully illustrates the dependence of mind upon matter, and likewise the facility of its ready response to impressions. Memory is that activity of the mind by which conceptions recur through association that were once present by perception. Two elements of necessity exist in mental exercises, and but two—the objective and the subjective. There is something to perceive, and something to perceive it with.

There are but two kinds of knowledge accessible to intelligence—the real and the ideal. Knowledge of the first is obtained by reason, of the second by imagination. Neither acquisition is possible without the indispensable influence of the objective. The perceiving capability is dormant, of necessity, without the tangible something to perceive.

If then, exercises of mind are impossible without the constant influence of physical agencies; and if every mental activity involves nervous waste; and if nervous waste may implicate general structural mutation; and if in this general structural lesion specific organs may be included; and if specific change may be sometimes morbid and abnormal, then is not the inquiry pertinent: may not mentality largely and insidiously determine disease or lesion of structure?

Light is a "mode of motion" whose impressions fall upon every surface. The surfaces thus affected must yield wholly, partially, or not at all. If wholly, the light is "white"; if partially and modified, one of the prismatic colors, determined by the form of the yielding; and if not at all, we have darkness. Color then is a "mode of motion" in light, depending upon the peculiarity of the reacting or reflecting surface. It is much the same with the mind's influence upon the body. To understand this statement and illustration fully, the absolute brotherhood of force must be understood and admitted.

It will be remembered that thinking and cerebral activity are practically interchangeable expressions. Every intellection of brain falls upon the entire organism, through our inevitable dynamic corelation, inducing either excessive, normal or deficient, added stimulation. The first and last dispose toward disease, the second supplies a waiting necessity. Instances verifying these propositions readily occur without reflection or enumeration.

Material and mental power present for consideration certain necessary qualities, without which we cannot conceive of the existence of either. The three elements of material power, or more appropriately, material existence, are extension, impenetrability, and inertia; of mental: perception, sensation and will. These last are given in every mental act. They are required to constitute it such. Eliminate one, and the mind's exercises are as effectually destroyed, as would be the constitution of matter without its essential characteristics. This fundamental identity of composition and requirements, is to me strongly presumptive of qualitative relationship. If this be indeed true, the all-pervading principle of the practical unity and correlation of phenomena, adds another to the accumulated evidence of universal organic connection with mental influence.

Another argument for the identities I claim, is derived from the simplicity of all force, as far as known. One illustration, amplified from the natural world, may suffice. The solar influence, one of whose phases is light, is not compound but simple. Its seeming composition is due to the varied peculiarities of the media through which the light passes and the surfaces against which it impinges. Differences in momenta determine differences in refrangibility. That one force should have several momenta is well nigh an inexplicable paradox. The problem is solved by the construction of the luminous wave. The phenomena of mind too, are simple. This no one can deny. The unity of human consciousness by which personal identity is uninteruptedly maintained, is possible only through unity of mental action. Every effort to reconcile the diversity of consciousness with the preservation of individual personality, has been a signal failure. All force is a related, conserved and dependent unit, and as just illustrated, affords still another confirmation of what my original proposition affirms. My argument must not be mistaken for a restatement of the doctrine of "reflex action." There is, of course, a mutual blending in some important particulars, but still they are fundamentally dissimilar. I am attempting suggestions relative to the development of mental power; and likewise to its controlling influence upon organization, as legitimate sequences of the correlation and conservation of force. Strictly speaking, I presume, all disease is wrought in consonance with one philosophy, and yet the circumstances attending its appearance and progress seem so diversified and conflicting, that well defined distinctions and definitions are required. In reflex action the mind is impressed and, at once, in the remote distributions of the nervous system, changes of tissue occur. This result is subsequent to organization. In the discussion I am indulging, mental action is contemplated in immediate relation to its mechanical equivalent—life, the builder of organization. An instance illustrating both, is the perturbation resulting in syncope. If the subjective cause of the first fainting could be prolonged and increased, commensurate with the degree to which the patient has become accustomed to it by experience, some obstinate and often fatal disease would supervene. This influence is cumulative by reflection. Its highest development is found in the subjective.

Another argument I offer may be called a novel one. I am not particular how they come, if their miscellaneous arrangement is not allowed to interfere with their appropriateness.

I refer to the economy and providence of nature in all its work. Whatever the human being needs, the human being is competent to supply, else his endowments are naturally and deplorably incomplete. Now, there is nothing we all so much require, as intelligent, discriminative power, to guide our bodies in safety amid all the dangers and vicissitudes to which they are unavoidably exposed. The power of mind is the only capability qualified to meet this requirement. It cannot do it at all without intimate and often controlling relation to the entire organization. If it is admitted that any such essential relation exists, and is exercised continually in normal relation to life and health, then it must likewise be conceded, that in the aberrations to which all force is subject, derangement and structural change may and so does occur. There is a large and important class of diseases, familiar to every practitioner, whose etiology is obscure. Professional ingenuity and acumen are exhausted in fruitless efforts to assign for their existence and variety, adequate causation. In such cases I suggest a thorough investigation of the mental influence involved, intimating that astonishing results are here in store for some one. This examination cannot be successfully made without an exact knowledge of the manifold science involved.

I may be permitted to add still another illustration.

My argument now shall be a series of contrasts between man and the lower animal and vegetable. My object is to attempt to show the transforming and cultivating tendency of mind upon organized beings. The vegetable, though a simpler order of life than the animal, of even the lowest kind, still has some common characteristics. The principle and phenomena of life are essentially the same. It is the fact of consciousness that gives the animal his distinctiveness and individuality. Not the conscious intelligence inert and dormant, but active, aggressive and self-reliant. Indigestion, respiration, assimilation and decay, the same general principles are observed throughout all organic creation. Anatomy and physiology are one in principle where the laws of organization prevail. Matter is of necessity immeasurably inferior to the animal, no matter how exquisitely its crystalline structure may be conformed, or how resplendent its beauty by the touch of art. The animal inferior to man is such from the deficiency of mental power. Man is afflicted by different diseases, adjusted to unlike offices, and experiences riper and richer feelings. The inquiry now pertinently arises: if man is thus different and superior to other animals in virtue of his perfect and complicated mental enrichment, which to so large an extent determines all his discipline, habits and emotions, may not these same rare gifts be essentially related to disease?

Again, it may be maintained that mental and vital forces are but cöordinate links in the one dynamic chain, from the variety of philosophy involved in their cultivation and growth. They each develope by exercise. This is the law and excellence of life and health. Every additional increment of power is gained only by paying it an industrious tribute. I receive it as a demonstration that a common principle of development establishes a common origin. Not only is this law of growth applicable in general, but in all special particulars likewise. The exercise, resulting in discipline, must be guided by the same rational requirements in both instances. Excess and deficiency are everywhere equally disastrous. The quality and opportunity must, with the same discreet foresight, be chosen. The only distinguishing feature claimed by intelligence, is the capability of independent volition. All who watch with care the characteristics of this element of mind, will see applied the same law and consequence of culture. Now I am disposed to the judgment, that so intimately are these forces related to each other, and to the same law of exercise, that deficiency or excess, as currently supplied, lead to frequent and obstinate disease. As long as the human organization is regarded as composite, respecting the existence of separate and independent entities—the material and spiritual, science must be, in degree, confused and unsatisfactory; but when all phenomena are received in their true light, as flowing from one common force, the millennial day of science will be ushered in. An argument, too, is founded upon the facts of the untiring activity of mind and the indestructibility of force. All mental action, not envisioned in consciousness, is consumed in influencing vital action. The phenomena that action and reaction are equal, is just as true of mind as matter. It really appertains to force rather than to either of these assumed entities. But few understand the meaning of this very common philosophy. A ball, for example, is thrown against a wall, and the reaction, determining the rebound, is equal to the original impulsion. Is it true we ask, after all, that the reflecting surface returns the force undiminished? As an obvious or mechanical fact, I answer no. When mechanical force seems to be expended, it is not lost, but changes its form, conserving every increment in the mutation. This is all that is meant by the proposition that action and reaction are equal. The hammer is not struck as hard as it strikes, but if all the divided motion excited by the hammer, were directed against appropriate machinery, the power exerted would be just equal to the first. The same principle prevails in mind. Every mental exercise is continued through the vicissitudes of material change. Much thought is signified in language, whose expression costs structural death and elimination, and much more, without being thus embodied, records the fact of its existence in the increased residuum of the emunctories. This suggestion surely recalls the intimate subjection of the physical to one of its forces—mind. A familiar and striking illustration of the power of mental influence in regulating and subduing the physical, is found in the causes that control age. Men do not grow old by becoming bald, gray, and by losing the elastic step, but by the loss of mental and social fire; by unseemly moroseness and failing urbanity. The cares and unhappiness of domestic life, of unsatisfactory business relations, these being yielded to by the intellect, soon induce the annoying symbols of age. If the mind is so gifted as to be superior to all these besetments, life must continue long and well. To constantly dwell upon any evil deplored, is almost the surest way to incur the affliction. Become practically oblivious to its existence, and life and health are at a premium. Every physician recognizes the aid afforded by a cheerful and contented mind in the administration of medicines. What most regard as an important therapeutic agent of cause, I am disposed to suspect as the cause of the disease. While my fellow-practitioners subordinate this auxiliary to medication, I shall subordinate all medication to it. While, in a certain class of cases, he enforces the versatility and variety of medicines, I shall enjoin an observance of the plain requirements of rational mental physiology. When revulsion is attempted by the infernal miseries of counter-irritation, I shall often secure a choice result by the revulsion of emotion. I shall check nasuea with a joy, dyspepsia with a hope, phthisis with a spree, and rheumatism with grit and warmth.

One of the most unexpected yet complete and beautiful illustrations of the conservation of force, is found in the equivalency of chemical and vital phenomena witnessed in the human body after death, and the imperceptible mutations transpiring during life. The first is purely obedient to inorganic chemistry, the last to vital action. In three and a half years the elimination of nitrogenized matter is equal to the sum of all such tissue in the normal body, and the dead body, when decomposition has been subject to certain conditions appropriate to the action of chemistry, will last before conversion into "adipocere" the same length of time. Do not these facts sustain the equivalent quantity of these forces, and likewise their essential brotherhood? It adds another to the largely accumulating lists of evidence establishing the compensations of all phenomena. The relation of mental influence to vital action is so intimate and indisputable, as not to excite the investigation its importance demands. The emotions are the best indices of the extent to which the mind is working. Intellections sometimes dissemble and mislead, the emotions are always truthful. These seem to be connecting links between the intellect and matter. They hold both in sympathy and support. No organ of the physical can remain long unperturbed in the focus of the feelings. Their burning and eloquent power soon disturb the otherwise even current of their life and health. These disturbances, long continued, are disease.

It seems that pathologists have proved that disease is structural leison, whose symptoms are indicated by functional derangement and final ruin, if repair be not speedily instituted and secured. The aptitude for lesion in any tissue, one would suspect, would be determined by the especial character of the tissue. This natural presumption is sustained by facts. It is known that nervous tissue is the last to suffer structural lesion. The superior integrity of this tissue is not only full of benevolence, but indicates, as well, the fact of the maintenance of this vehicle of thought, against the mutations incident to organization in general. These, in connection with other facts, prove incontestibly the supremacy of mind over matter, and likewise the natural relation of the mental influences to the direction and control of organization. If it be denied that the brain and nervous system think and feel themselves, it will still be admitted that these are the organs through which thought is manifested. In either event, the point is sustained, that the mind's integrity is longest maintained, and its controlling relation to organization most intimate and persistent. Now the exercises of mind are largely subject to individual control, so that our life and health are in an important degree committed to the keeping of our volition. More, than with any other agency, reposes our life and health with our choice. Gloom and foreboding terminate in disease; cheerfulness and occupation are the harbingers of long and happy life.

If there be two entitities, mind and matter, the ultimate conditions of their being are not very discrepant. They must, in the dimness of practical infinity, approximate an absolute identity. This I infer from the divisibility of matter. There are those who object to this proposition, and affirm the infinite indivisibility of the ultimate atom. Of the existence of ultimate atoms there is not one item of experimental or conceptional proof. As long as man can mechanically divide matter, its ultimate indivisibility is not established. If a man divide a portion of matter in his hand, a portion must remain. Every fresh separation still leaves a portion in hand. When the division has gone so far as to be practically impossible any further, a part of the original piece is still intact. One's conceptions are only the antecedents of one's knowledge. Our conceptions are experimental possibilities to the incoming generation of advanced culture. An indivisible atom is then really inconceivable. To form a notion of an atom certainly three things are necessary, if we ascribe to it materiality: extension, impenetrability and inertia. What then is an atom? A unit certainly, whose half is as readily conceived of as its whole. There can then be nothing ultimate, and yet the approach to it must be infinite. Conservation and correlation are now the watchword, not ultimateness and eternity. Infinite divisibility in matter is requisite to condition impenetrability, without which no conception of matter is possible.

What I wish these remarks to subserve, is what I chose to call the progressive ultimateness of matter and mind. Concerning the simplicity of mind there is no controversy; the practical simplicity of matter is partially understood and conceded. In the mutual approach of these toward infinity I detect mutual harmonies and relationship. I find too, after these elements of existence coalesce in simplicity, the grandeur of volition transpires in assuming charge of all. To the enterprise and intelligence of the human will all ultimate existence is finally committed. Here, in this apparently remote relation to organization, I find the human mind moulding influences finally destined to confer disease or death. That such intelligent and conscious empire should exist, may not be deemed even strange when the necessity of it is once appreciated. The elaborate and insentient perfection of all organization requires the presence and guidance of an intelligent and capable directing agency. Such requirement the mind supplies. Its easy control has existed so long and behaved so well, that we are usually oblivious to its existence and insensible to its claims. The finest illustration of the influence of mind upon body, just now occurring to me, is found in the history of war prisons during our recent eventful and unhallowed war.

It was mind here that wrought the physical disease.

The altar of despondency claimed nearly all the sacrifices.

What was so palpably true in this horrible instance, exists as veritably in most diseases.

Any chemical element, compound or mixture, uninfluenced by life, is inorganic. Any chemical compound influenced by life is organic, and every mechanical mixture determined by life is an organization. The vital relation of life to disease and health, is concisely and definitely stated by these propositions, whose accuracy can hardly be assailed. A variety of circumstances transpire to illustrate the intimate dependence of normal life upon normal intellect and emotion. That life is the antecedent of every vital function, is undeniable. Are not these instances of mind affecting life, and through it of necessity all organization?

Bad news, long continued and often repeated; severe and irreparable disappointment; high and exalted hopes blasted forever in a moment; long and anxious foreboding over imaginary disaster; ever anticipated failure in business relations or domestic; the early and final ruin or death of darling love and hope; monomaniac devotion to any cherished work or thought; upbraiding and remorseless conscience; an intellectual zeal or religious enthusiasm untempered or uncontrolled by the strong arm of reason; loss of self-respect through general social ostracism; an unrequited and hopeless effort to be what is impossible. Are not all these, and immeasurably more, among the depressing agencies that subtract from the continuance of life.

It is the function of the brain to think, as it is of the eye to see or the ear to hear. It is fashionable with psychologists to assign to an imaginary entity, intangible and spiritual withal, absolute dominion over all instances of perception. This is a figment of scholasticism rather than a sequence of induction. "How shall we reason but by what we know?" Philosophy and common sense are really not far asunder. By the last all aspiring criteria must be adjuged. Common sense is that rare and valuable gift by which we accept what seems evident, without involving philosophic or linguistic twaddle to mystify and mislead. The eye seems to see, the ear to hear, and the brain to think. This is sufficient. It is the organ and its appendages that constitute its unity and entirety. If the nerves of special sense be severed, the organs of which they are physiologically integers are likewise destroyed. The integrity of every part is requisite to give the whole completeness and function. To say that the spiritual element in man, or even his brain, is the appropriate and exclusive means of perception, is an assumption purely gratuitous and unphilosophical, because unwarranted by what we know. That the exercises of brain exert an influence, either salutary or injurious, over the general organization, and thus play an important part in health and disease, seems to be universally admitted and utilized.

I cannot dismiss the presentation of the philosophies of this discussion, without recounting what I esteem the primal and positive evidence of unity of principle in matter and mind. Of course I allude to the indestructibility of the properties and forces of each. In matter we usually denominate this imperishable persistence, indestructibility; in mind, immortality. Both are true in virtue of reciprocal and interchangeable conversion,—the underlying and happy agency of all causation. I am sure no other reason has been assigned equally plausible, to account for or to establish the hope of immortality. The reasoning is simple and apparent. In the conversion of all force, constant equivalency is maintained. The phenomena of mind, being forms of force, are likewise conserved exactly amid all the vicissitudes to which their multiplied phases are exposed. This fact being no longer in doubt, how the extinction of the thinking principle at death can be for one moment maintained, is to me very singular. If the essential perpetuity of mind be like that of matter, can it be deemed mysterious, or impossible, that they should very intimately influence each other during the present life of both? When the conditions of existence and activity, now appertaining to each, and in virtue of which they so reciprocally interchange, are dissolved in death, then under new and perhaps unlike forms, the quantity of mental power must be preserved. What is true in death is equally true in life, and more evident.

An organism is a style of mechanics determined by life, and whose compound associated function is reproduction and death. For some reason there are indeed the law and excellence of life and growth. It is a fact, disputed by no one, that these normal and necessary metamorphoses are a prime and obvious sequence, in no unimportant degree, of well directed and persistent mental and moral action. No man is really healthy unless he be a thoughtful and industrious student. For every intellection there is a definite amount of nervous change; and for every natural nervous change there is made a definite contribution to vitality. Thinking is the exercise in which the nervous system delights to find health and pleasure. There is a great difference between thought, as it should be, and those mental gymnastics in which so many "educators" direct their pupils to indulge. Of course to such games of intellectual expertness I am not now referring. I allude to that calm, sedate, persistent mental exercise, in which the nervous system, and through it the entire organization, is inspired to normal nutrition. That the intellect and emotions are thus made almost the arbiters of life and death, seems an interesting and desirable truth. Intellectual and ignorant people are not subject to the same classes of disease. What are called "nervous diseases" never appertain in many of their phases to the illiterate and squalid.

Having enumerated, in detail, the circumstances under which mental influence may determine "physical" disease, it remains to specify some of the evidences diagnostic of the difficulties to which I refer. I am inclined to the judgment, that even where the lesions are palpable and their cause is evident, that certain investigations should be made, to decide with intelligence relative to the quantity and kind of the mental action involved. The physician may well be proud of his high distinction. He has access to the usually hidden and unintelligible philosophies of life and living. Hardly a scientific inquiry can be impertinent or irrelevant, and all must respond to his appropriate questions. I can mention no specific or formula by which to conduct these examinations. General considerations only are possible in the absence of any particular disease. The few I mention may hardly be deemed inappropriate. It will be understood that I am not excluding, in the connected questions I ask, the customary ones proposed to diagnosticate all the features of the malady, I attempt simply to systematize certain general methods of arriving at the influence of mind in every case of disease. The usual symptoms of particular disease are invaluable to elucidate the inquiry I propose to make. If sufficient importance is attached to what I offer, I care not what else is included. Unity and perfection are the prime requisites of medical examination. My first inquiry would be addressed to the general physical constitution of the body, with reference to its influence upon the mind. The predisposing tendencies of mind affecting organization may be, and usually are, determined by the general characteristics of the body; the quality and texture of nervous matter; the extent of fatty deposits; the kind and developement of muscle; the nature and variety of physical idiosyncrasy; the best average of appetite; the amount and quality of food eaten; the quantity and value of excreta; the extent to which the illness has been protracted, also the severity, and the degree to which it has been borne: these are physical considerations, never to be overlooked, in every medical examination for the extent and phases of disease. Again, the mind itself requires your care. What are its prominent charteristics? Is the mind "well balanced," or are there well marked latent or active special gifts or deficiencies? Has it seemed to hold empire over the vital functions, or to be naturally subordinate to them? What are its normal tendencies toward activity? Is it working most of the time, or only periodically, if at all? To what extent is emotion included in the mental action, and what peculiarities does it exhibit? How about the element of "will"? Is the mind resolute or yielding? How are good and bad fortune generally borne? Under what circumstances does the man generally give up when in health? These questions deduce his mental physiology.

Another class of questions should contemplate the patient's antecedents. Have they been favorable to the developement of mental power? Have his parents been well formed intellectually, and have they been accustomed to think and feel? Does the man resemble one or both of his parents, or even some distant relation, it may be, whose peculiarities may have been transmitted and retained? How about the man's cultivation; was he the victim of early and earnest study and discipline? In what has his mental training consisted? What kind of thought has been his preceptor? At what period of life did he begin to think, and what has been the extent to which in the immediate past it has been carried? What has been the moral education of the man, and has he been sensitive to its tendency? How has conscience been treated, and how has it behaved? Has the man been harrassed by its peculiar and unrelenting torment, or has his life been quiet and morally peaceful? To what religious ideas does he stand committed, and have any such been heartily embraced? If so, have they been liberal and cheerful; or dark, forbiding and hopeless? To this point especial attention should be directed. How about social and domestic affairs; is the man happily situated at home; if not, what elements are averse, and who is really most aggrieved? Are the patient's circumstances easy, or is he annoyed by pecuniary liabilities? These include almost an exhaustive enumeration of questions for general guidance and aid. An inquiry of prime importance is now suggested. What remedial measures are appropriate and available in this particular case? Of course nothing specific can be here mentioned. The entire treatment must be measurably synchronous and thoroughly consistent. The physical lesions, involved by other agencies and complicated by unknown influences, constantly modify every instance of disease. This must be borne in mind by the educated physician, whose reason is observing the phases and virtues of mental action. Nothing can be done without an intimate knowledge of mental physiology. A profound acquaintance with the mind, in action, is as necessary to rational psychological therapeutics, as the familiar physiology of life is to ordinary medicine.

The indispensable knowledge of physiology is the thing most required to qualify the practitioner for his honest duties. Medicine is empiricism without it, and cures an accident. The normal deportment of mind being known, every pathological aberration is understood. The mind requires appropriate stimulation like the body. From its peculiar pabulum it must be nourished. Mental disease is mal-mental nutrition. It is the duty of the practitioner to ascertain and study any deficiency or excess in this direction, and meet them, in harmony with the demand of nature. I must forbear. The subject is still interesting, and seems exhaustless. Every medical college should teach the science of the mind, with especial reference to disease and remedy.

